# Pimozide-induced tardive dyskinesia in the treatment of delusions of infestation

**DOI:** 10.1016/j.jdcr.2024.01.010

**Published:** 2024-01-20

**Authors:** Rodrigo A. Gutierrez, Payton Smith, Allison Kranyak, Mitchell Davis, John Koo

**Affiliations:** a School of Medicine, University of California, San Francisco, California; b Department of Dermatology, University of California, San Francisco, California

**Keywords:** Delusions of infestation, pimozide, psychodermatology

## Introduction

Delusions of infestation (DOI) is one of the most challenging disorders that dermatologists will encounter in their practice. One of the most widely used and studied agents in the management of DOI is pimozide.^1^ Pimozide has unique utility in the United States in that it has no psychiatric indication per the Food and Drug Administration, despite bearing structural resemblance to antipsychotics. Patients with DOI are generally resistant to accept psychiatric referral or to take any medication officially labeled as an antipsychotic. Thus, pimozide is frequently the only medication with antipsychotic efficacy that DOI patients are willing to take.^2^

Like antipsychotics, pimozide carries the risk of tardive dyskinesia (TD). Fortunately, the occurrence of TD from pimozide therapy in dermatology appears to be exceedingly rare.^2^^,^^3^ Herein, we describe a case of TD in dermatologic use of pimozide.

## Report of case

A 52-year-old woman with no psychiatric history was referred to the University of California, San Francisco dermatology clinic in 2012 for a diffuse, painful skin sensation over the scalp that the patient described as “things gushing out like a fountain.” Her symptoms first occurred several months prior during a home remodeling project. She reported “strings” and “fibers” that started “beneath the skin” and eventually “protruded through the surface of the skin.”

She had previously completed 2 courses of oral cephalexin prescribed by an outside provider which reportedly “caused the symptoms to spread” across her face and body. Culture of the lesions was consistent with impetigo, and she subsequently completed several months of minocycline therapy without improvement. Her symptoms were occupationally and socially impairing, resulting in near-total isolation and the loss of her job as a business executive.

Initial physical examination revealed only excoriations on the face, scalp, and neck. Subsequent laboratory workup, including complete blood count, thyroid function tests, B12 levels, liver function tests, and electrocardiogram, revealed no significant findings. The patient was started on oral pimozide at 2 mg daily with a plan to up-titrate by 0.5 mg every 2-3 weeks to reach 3 mg daily, a dose that is usually effective for this condition.^2^

**Fig 1:**
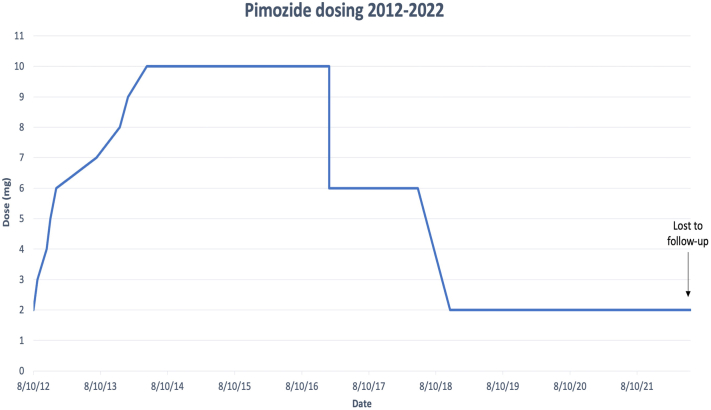
Adjustment of pimozide dosing over 10-year treatment course

The patient consistently took oral pimozide at increasing doses for a period of nearly 2 years (Fig 1). After she failed to respond to 3 mg daily, her dose was gradually increased due to lack of symptom control to 10 mg, at which time the patient finally experienced significant improvement. At this point, the patient experienced periods of remission which gradually increased in duration, allowing the patient to maintain an occupation.

Despite taking pimozide at 10 mg daily, the patient had experienced only mild side effects of akathisia that were rapidly alleviated with as-needed doses of 2 mg of oral benztropine. However, any attempt at lowering the dose of pimozide was met with worsening of DOI symptoms. The patient was maintained on 10 mg daily for 2 years.

Due to concern regarding the risk of a high dose of pimozide, a switch to risperidone was attempted. However, upon switching, the symptoms briskly returned, requiring a rapid return to pimozide.

After 1 additional year at 10 mg, the patient reported symptom-free periods lasting several months. Her dose was successfully reduced to 6 mg daily for 2 years, followed by another reduction to 2 mg daily, a dose at which her condition appeared to stabilize. Despite pimozide exposure of over 10 years, the patient did not demonstrate serious adverse effects, despite checking for TD at every follow-up visit.

The patient then sought advice from a neurologist to better control her mild, intermittent flares. During her visit, very mild buccolingual dyskinesia and head titubation, consistent with tardive dyskinesia, were noted on physical exam. This prompted the discontinuation of pimozide, which exacerbated her delusions and hallucinations. However, the TD itself was deemed by neurology to be too mild to warrant treatment. In total, the patient had received a cumulative dose of 20,418 mg of pimozide over her ten-plus years of treatment.

The patient was prescribed 25 mg daily doses of quetiapine as a replacement for pimozide, with a plan to increase the dose gradually as tolerated. However, the patient firmly felt that pimozide was the only effective agent, and that her condition was not psychological, but exclusively due to a physical, cutaneous etiology. She eventually became lost to follow-up and obtained a pimozide prescription from different providers.

## Discussion

Given over 10 years of treatment with more than 3 years at a 10 mg daily dose, this case distinguishes itself in its prolonged duration of treatment at a high, sustained dose compared to typical DOI patients treated with pimozide. For more than 3 decades, the author’s (J.K.) experience has been that typical DOI patients respond to pimozide at doses of around 2-3 mg/d and can be tapered off without recurrence after a few months.

Therefore, there are 2 notable features of this case. First, it exemplifies the rarity of TD from pimozide in dermatologic use and underscores the high doses and prolonged duration of therapy typically needed to develop TD. The second notable feature of this case is that it illustrates how some patients can become very emotionally invested in pimozide once they experience benefit, despite often being ambivalent when they first agree to take it.

It is important to note that if a patient treated with pimozide develops TD, a neurologist is best equipped to manage this side effect. In addition to stopping the offending agent, TD can be treated pharmacologically with valbenazine and deutetrabenazine.^4^^,^^5^ These agents inhibit the synaptic overactivity of dopamine believed to contribute to TD symptoms. Features that distinguish TD from drug-induced parkinsonism include delay in onset and arrhythmic, choreo-athetoid movements typically involving the face.^6^

This report illustrates a rare occurrence of TD with the use of pimozide in treating DOI. To the best of the authors’ knowledge, this case is only the second case of TD to be reported in the medical literature from the use of pimozide in dermatology. This side effect occurred after the patient experienced substantial alleviation of DOI symptoms at high doses of pimozide for prolonged periods of time.

## Conflicts of interest

None disclosed.
